# Ameloblastin Peptides Modulates the Osteogenic Capacity of Human Mesenchymal Stem Cells

**DOI:** 10.3389/fphys.2017.00058

**Published:** 2017-02-07

**Authors:** Øystein Stakkestad, Ståle P. Lyngstadaas, Jiri Vondrasek, Jan O. Gordeladze, Janne Elin Reseland

**Affiliations:** ^1^Department of Biomaterials, Institute of Clinical Dentistry, University of OsloOslo, Norway; ^2^Department of Bioinformatics, Institute of Organic Chemistry and Biochemistry, Czech Academy of SciencesPrague, Czechia

**Keywords:** ameloblastin, biomineralization, bone growth, exon 5, human mesenchymal stem cells, osteogenesis, proliferation

## Abstract

During amelogenesis the extracellular enamel matrix protein AMBN is quickly processed into 17 kDa (N-terminus) and 23 kDa (C-terminus) fragments. In particular, alternatively spliced regions derived by exon 5/6 within the N-terminus region are known to be critical in biomineralization. Human mesenchymal stem cells (hMSC) also express and secrete AMBN, but it is unclear if this expression has effects on the hMSC themselves. If, as suggested from previous findings, AMBN act as a signaling molecule, such effects could influence hMSC growth and differentiation, as well as promoting the secretion of other signaling proteins like cytokines and chemokines. If AMBN is found to modulate stem cell behavior and fate, it will impact our understanding on how extracellular matrix molecules can have multiple roles during development ontogenesis, mineralization and healing of mesenchymal tissues. Here we show that synthetic peptides representing *exon 5* promote hMSC proliferation. *Interestingly*, this effect is inhibited by the application of a 15 aa peptide representing the alternatively spliced start of *exon 6*. Both peptides also influence gene expression of RUNX2 and osteocalcin, and promote calcium deposition in cultures, indicating a positive influence on the osteogenic capacity of hMSC. We also show that the full-length AMBN-WT and N-terminus region enhance the secretion of RANTES, IP-10, and IL-8. In contrast, the AMBN C-terminus fragment and the exon 5 deleted AMBN (DelEx5) have no detectable effects on any of the parameters investigated. These findings suggest the signaling effect of AMBN is conveyed by processed products, whereas the effect on proliferation is differentially modulated through alternative splicing during gene expression.

## Introduction

Ameloblastin (AMBN) is an extracellular matrix protein expressed in mesenchymal and epithelial cells (Fong et al., 1998). Epithelial-mesenchymal interactions initiate tooth development and has been shown to induce the expression of AMBN (Takahashi et al., 2012). AMBN is also involved in biomineralization in other tissues than teeth, and is expressed and secreted from cultured human mesenchymal stem cells (hMSC) and osteoblasts (Tamburstuen et al., 2011). Expression of AMBN (Spahr et al., 2006; Tamburstuen et al., 2010) in cells bordering bone defects suggest a role for AMBN in the recruitment, growth and differentiation of hMSC, and is a potential target for clinical interventions for bone healing.

AMBN is known to modulate proliferation and differentiation of ameloblasts, periodontal ligament cells (PDL), pulp cells (Nakamura et al., 2006) and hMSC; (Fukumoto et al., 2004; Sonoda et al., 2009; Tamburstuen et al., 2010; Zhang et al., 2011). AMBN splice variants are widely distributed in time and location, and have several roles during dental biomineralization. During early tooth development in mice, the predominant splice variant is 15 aa shorter than the splice variant expressed in later stages. These 15 aa (*Q9NP70)* derive from the start of *exon 6* (Cerný et al., 1996; Fong et al., 1996; Hu et al., 1997; Lee et al., 2003; Ravindranath et al., 2007). However, spatial distribution and post-translational modification of the splice variants present in mesenchymal tissues like bone still needs to be investigated.

Extracellular full-length AMBN has not been identified *in vivo*. This is most probably due to rapid degradation by co-secreted specific matrix metalloproteases like MMP-20 (Uchida et al., 1997). Thus, it is important to understand the dynamics of AMBN processing and the biological role of the processed products. It is interesting that recombinant full-length AMBN has been found to enhance the secretion of cytokines and chemokines involved in inflammation and recruitment of progenitor cells (Tamburstuen et al., 2010). This effect is evident in the healing of critical-size defects in the jawbone of rats (Spahr et al., 2006; Tamburstuen et al., 2010) and in pulpal wound healing in pigs (Nakamura et al., 2006). These findings suggest that the full-length product has a biological (or at least pharmacological) effect on its own or that the full-length molecule act as a founding source for shorter, active, peptides.

Unprocessed ameloblastin (AMBN-WT) is a two-domain protein where the amino- and carboxyl ends are differently organized with opposing chemical properties (Vymetal et al., 2008). The N-terminus is defined by the first 10 exons encoding 222 aa (human). Both the unprocessed ameloblastin and the N-terminus may form fibrils through self-assembly supported by the *exon 5* derived region (Wald et al., 2013). The C-terminus is defined by the last three exons encoding 225 aa (Toyosawa et al., 2000). It has been suggested that parts of the porcine N-terminus (17 kDa fragment) is active in mineralization and regeneration (Fukae et al., 2006; Stout et al., 2014), whereas the C-terminus product may have a role in cell surface attachment (Sonoda et al., 2009).

*In silico* modeling of AMBN-WT folding, suggests that some discrete peptide-sequences are exposed on the surface of the folded protein structure (Vymetal et al., 2008). It is reasonable to assume these domains have biological functions that are presently unknown. Whether these domains act in concert or have individual activities also need assessment. Based on *in silico* modeling, we have designed synthetic peptides from exons 2–13 (without signal peptide) representing these exposed domains. In an attempt to look for biological effects of AMBN and the processing products as suggested by Tamburstuen et al. and others (Nakamura et al., 2006; Spahr et al., 2006; Tamburstuen et al., 2011), these peptides and various other AMBN fragments were added to cultures of hMSC, and the cells were monitored for changes in growth and differentiation as well as effects on levels of selected cytokine and chemokine secretion.

## Materials and methods

### Experimental design

Human mesenchymal stem cells (hMSC; Cat.no: PT-2501, Lonza Walkersville, MD, USA) were maintained in growth medium [GM; Cat.no: PT-3238 supplemented with MSCGM SingleQuots, Cat.no: PT-4105, Lonza (http://www.lonza.com/products-services/bio-research/stem-cells/adult-stem-cells-and-media/human-mesenchymal-stem-cells-media.aspx)] and changed every 3rd day. These hMSC cells are isolated from normal (non-diabetic) adult human bone marrow withdrawn from bilateral punctures of the posterior iliac crests of healthy volunteers.

**Figure 1:**
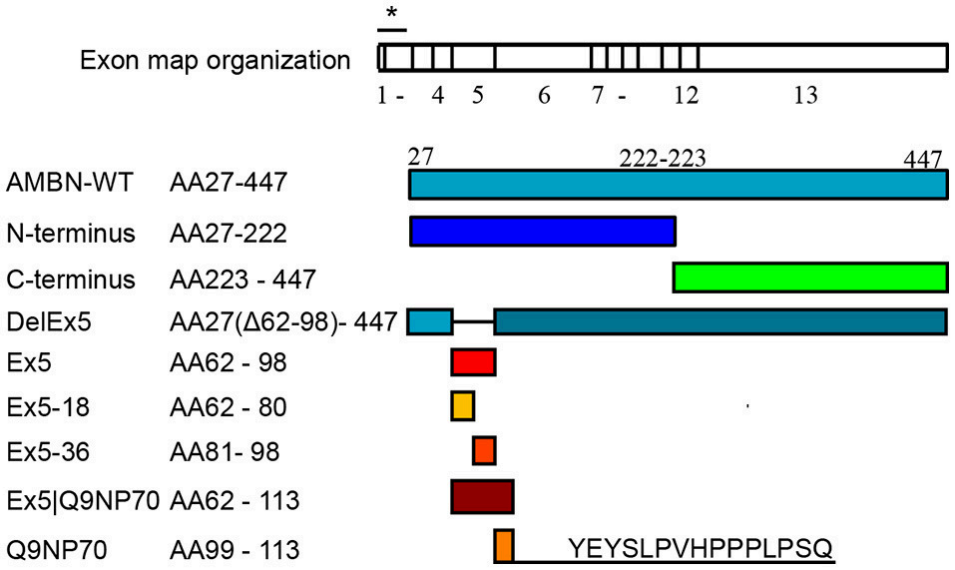
**Overview of recombinant ameloblastin (AMBN) proteins and derivatives of the N-terminus region**. Signal sequence of the 26 amino acids is indicated with asterisk in the exon map that is scaled according to the stretch of residue length in each exon.

The recombinant AMBN protein, fragments, and peptides, presented in Figure 1, were produced and purified as described by Wald et al. (2013).

Human MSC were incubated with 0.1 μM and 0.2 μM of AMBN-WT, AMBN-WT without Exon 5 (DelEx5), N-terminus of AMBN, C-terminus of AMBN, or 0.2 μM of exon 5 related peptides [Ex5 (AA62-98), Ex5-18 (AA62-80), Ex5-36 (AA81-98), Ex5|Q9NP70 (AA62-113), and Q9NP70 (AA99-113)]. Untreated hMSC were used as control at each time point tested. Cells and culture medium were harvested after 1, 3, 7, 14, 21, and 28 days of incubation.

### Proliferation assay

hMSC (6000 cells/well) were seeded in 48 well plates and incubated with the various test fragments or controls for 24 h. New DNA produced in the cells was labeled with 0.1 μCi [^3^H]-thymidine in a 12 h pulse prior to harvesting. The cells were then washed twice in PBS and then twice in 5% TCA to remove excess thymidine, and the remaining pellet dissolved in 1 M NaOH. Optifluor Scintillation liquid (Lumagel LSC GE BV, Groningen, Netherlands; 4 ml) was added, and radioactivity was measured in a Packard 1500 TRI-CARB liquid scintillation counter (Perkin Elmer, Shelton, CT, USA).

### Measurements of secreted biomarkers

Harvested cell-culture-medium samples were concentrated 5-fold in spin columns with a 3 kD cut-off (Pall Life Science, Ann Arbor, MI, USA).

The concentrations of Eotaxin, granulocyte-colony stimulating factor (G-CSF), interferon (IFN) α2, IFNγ, interleukin (IL)-1α, IL-1β, IL-1 Receptor Antagonist (RA), IL-2, IL-4, IL-5, IL-6, IL-7, IL-8, IL-10, IL-12, IL-13, IL-15, IL-17, interferon gamma-inducible protein (IP-10), monocyte chemoattractant protein-1 (MCP-1), macrophage inflammatory protein-1 alpha (MIP-1α), macrophage inflammatory protein-1 beta (MIP-1β), regulated upon activation normal T-cell expressed and secreted (RANTES), tumor necrosis factor alpha (TNFα), vascular endothelial growth factor (VEGF), as well as adreno-corticotrophic hormone (ACTH), Dickkopf-1 (DKK1), Insulin, Leptin, osteoprotegerin (OPG), osteocalcin (OCN), osteopontin (OPN), sclerostin (SOST), fibroblast growth factor-23 (FGF-23), respectively, were measured using HCYTOMAG-60K and HBNMAG-51K assays (MILLIPORE corporation, Billerica, MA, USA), respectively, and analyzed with the Luminex xPONENT version 3.1.871 or MILLIPLEX™ Analyst version 5.1 software in the Luminex-200 system (Luminex Corp., Austin, TX, USA). Only the cytokines and chemokines that showed significant change are discussed here.

### mRNA isolation

hMSC were washed in PBS and lysed, and the mRNA was isolated using magnetic beads according to manufacturer's instructions (Dynabeads Oligo (dT)_25_, Life Technologies, Gaithersburg, MD, USA). The mRNA was separated from the beads by heat treatment (80°C for 2 min), and quantified using a nano-drop spectrophotometer (ND-1000, Thermo Scientific, Wilmington, DE, USA, with software version 3.3.1.).

### Real time PCR

cDNA was generated from mRNA using Revertaid First Strand cDNA synthesis kit (Fermentas, Burlington, Ontario, Canada) according to the producer's instructions. Real time PCR was performed using Ssoadvanced SYBRGreen Supermix (Bio-rad, Hercules, CA, USA) in a reaction mix of 20 μl (1 ng cDNA) in 96 well plates using the CFX Connect™-system. Gene expression was normalized to reference housekeeping genes β-actin and glyceraldehyde phosphate (GADPH) using the ΔΔCT method with Bio-Rad CFX Manager software version 2.1. The primer sequences used are listed in Table 1.

**Table 1:** **Description of human primers used in experiments**.

**Genes**	**Forward primer**	**Reverse primer**
OCN	5′-GAAGCCCAGCGGTGCA	3′-CACTACCTCGCTGCCCTCC
Col1α1	5′-AAGGGACACAGAGGTTTCAG	3′-TAGCACCATCATTTCCACGA
RUNX2	5′-CCAGATGGGACTGTGGTTACC	3′-ACTTGGTGCAGAGTTGAGGG
Osterix	5′-GCCAGAAGCTGTGAAACCTC	3′-GCTGCAAGCTCTCCATAACC
β-Actin	5′-CTGGAACGGTGAAGGTGACA	3′-AAGGGACTTCCTGTAACAATGCA
GADPH	1′–TGCACCACCAACTGCTTAGC	2′-GGCATGGACTGTGGTCATGAG
RANKL	5′-CGGGGTGACCTTATGAGAAA	3′-GCGCTAGATGACACCCTCTC
OPG	5′-TGGGAGCAGAAGACATTGAA	3′-GTGTCTTGGTCGCCATTTTT

*OCN (osteocalcin), Col1α1 (collagen type 1 α1), RUNX2 (Runt-related transcription factor 2), GAPDH (glyceraldehyde 3-phosphate dehydrogenase), RANKL (Receptor Activator of Nuclear Factor κ B), OPG (osteoprotegerin)*.

### Mineralization

Cells were cultured to confluence in 12-well plates and then treated with AMBN or its fragments in either GM or osteogenic differentiation media (DM; Cat.no: PT-3924, supplemented with hMSC osteogenic SinglequotsTM) for up to 28 days. Medium was changed every 3rd day. Upon harvest, the cells were washed three times with PBS, fixed in 95% ethanol for 30 min, and then stained with 1% alizarin red for 5 min as described elsewhere (Dahl, 1952). To quantify mineralization, the alizarin red deposition was extracted with cetyl pyridinium chloride (Sigma-Aldrich, St. Louis, MO, USA) at room temperature, and measured at 562 nm in (EL × 800 Absorbance Reader, BioTek instruments, Winooski, VT, USA).

### Statistics

Student *t*-test was used to evaluate the effect of AMBN and its fragments compared to untreated controls at each individual time point. The Mann-Whitney U- Test was used if the results were not normally distributed. The significance level was set to *P* ≤ 0.05.

## Result

### Proliferation of hMSC is influenced by peptides derived by *exon 5* and *Q9NP70*

**Figure 2:**
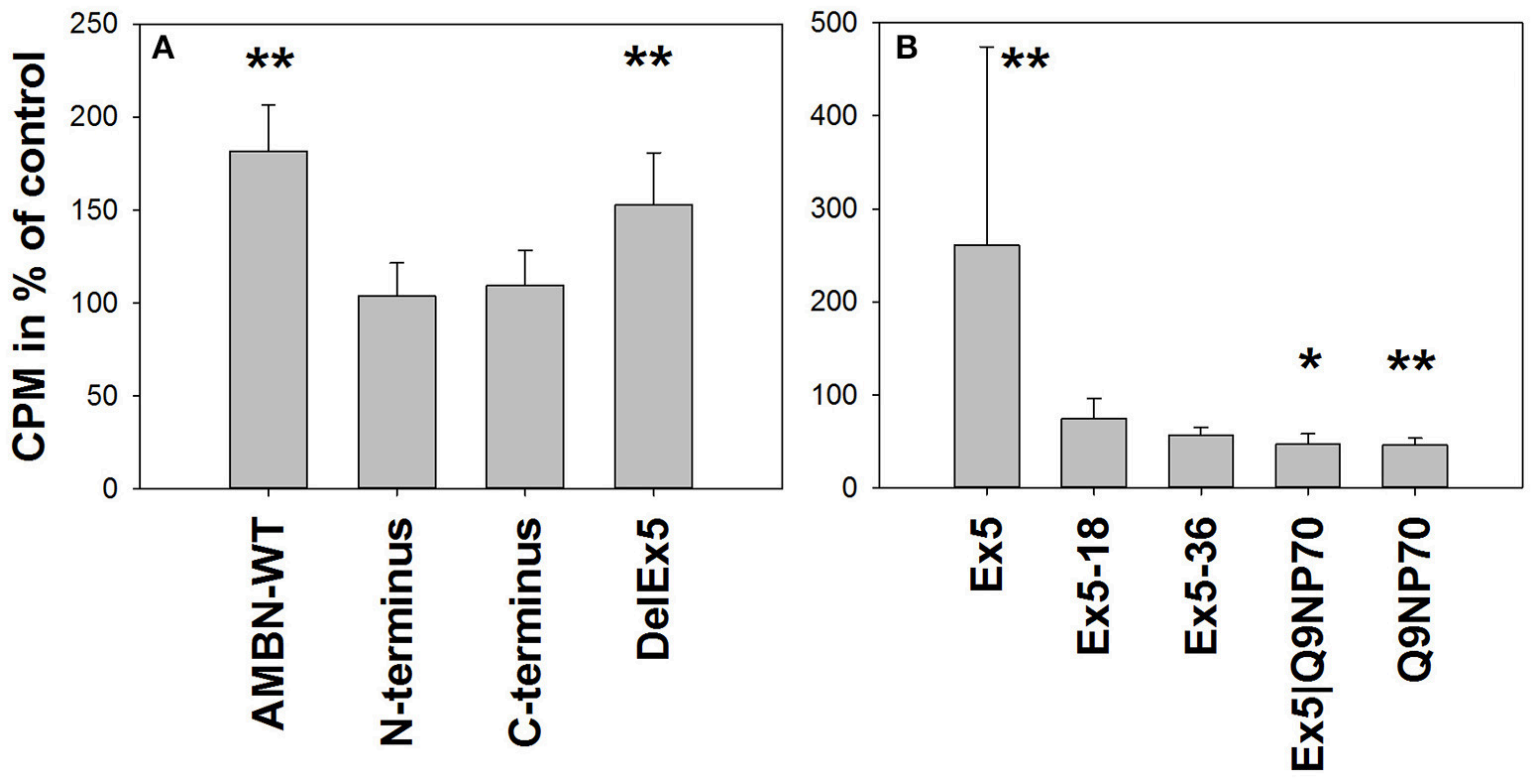
**Proliferation measured as [^**3**^H]-thymidine incorporation in hMSC exposed to (A)** 0.1 μM AMBN-WT, N-terminus, C-terminus or DelEx5 (*n* = 6). **(B)** 0.2 μM Ex5, Ex5-18, Ex5-36, Ex5|Q9NP70, or Q9NP70 (*n* = 12). ^3^H-thymidine incorporation (CPM) was calculated relative to untreated control cells (%), and presented as mean ± *SD*. ^*^Indicate *P* < 0.05, ^**^Indicate *P* < 0.01.

AMBN-WT (0.1 μM) enhanced cell proliferation of hMSC to 1.8-fold (*P* = 0.002) of the control. DelEx5 was found to enhance proliferation to 1.5-fold (*P* = 0.004), whereas no effects were observed from the C-terminus or the N-terminus fragments alone at the time points tested (Figure 2A). Among the tested peptides, Ex5 enhanced the proliferation to 2.6-fold (*P* = 0.004) while Q9NP70 and Ex5|Q9NP70 both inhibited proliferation to 0.5-fold of control (*P* = 0.015 and *P* = 0.003, respectively; Figure 2B).

### Cytokine and chemokine secretion is enhanced by AMBN-WT and N-terminus

AMBN-WT (0.2 μM) enhanced the secretion of RANTES 19 and 21-fold at day 7 and 14 respectively (*P* = 0.015 and *P* = 0.019). The secretion of IP-10 was enhanced 22-fold and 24-fold at day 1 and day 14, respectively (*P* = 0.029 and *P* = 0.047). Finally AMBN-WT enhanced the secretion of MIP-1α 8-fold at day 1 (*P* = 0.043) and 12-fold at day 14 (*P* = 0.001; Table 2). Lower concentration (0.1 μM) of AMBN-WT produced only a slight increase in secretion of MCP-1 and IL-6 at day 1 (values not shown).

**Table 2:** **Cytokine/chemokine secretion from hMSC**.

**Chemokine**	**Days**	**AMBN-WT**	**DelEx5**	**N-term**	**C-term**
RANTES	1	10 ± 6	1.5 ± 0.8	2.5 ± 1.3^*^	0.9 ± 0.2
	3	0.9 ± 0.4	0.7 ± 0.04	1.3 ± 1	0.9 ± 0.4
	7	19 ± 7^*^	1 ± 0.3	4.5 ± 0.6^***^	1.3 ± 0.3
	14	21 ± 9^*^	1 ± 0.5	3 ± 0.7^**^	0.9 ± 0.3
IP-10	1	22 ± 13^*^	2 ± 1	5.5 ± 0.8^**^	1 ± 0.4
	3	1.6 ± 0.08	2 ± 0.2	1 ± 0.5	2 ± 0.3
	7	24 ± 14^*^	1.5 ± 0.4	5.8 ± 5	1.7 ± 1
	14	21 ± 7.9	1.8 ± 0.7	2.7 ± 0.5^*^	1.2 ± 0.3
IL-8	1	2 ± 1.3	1.6 ± 113	2.3 ± 2.1	1 ± 0.8
	3	3 ± 0.5	1.3 ± 0.4	2.5 ± 1.3^*^	1.2 ± 0.04
	7	4 ± 0.8	1.7 ± 0.6	3.9 ± 0.8	1.6 ± 0.6
	14	3 ± 0.6	2 ± 0.7	3 ± 0.3	1.7 ± 0.6
MIP-1α	1	8 ± 5.5^*^	1 ± 0.5	2.4 ± 1.3	0.7 ± 0.4
	3	n/a	n/a	n/a	n/a
	7	14 ± 6	3 ± 0.6	4.7 ± 0.7	1.5 ± 0.05
	14	12 ± 2^***^	1.3 ± 0.6	2.6 ± 0.3^**^	0.8 ± 0.1

*Secretion of RANTES, IL-8, IP-10, and MIP-1α from human mesenchymal stem cells (hMSCs) grown in standard growth media containing 0.2 μM of AMBN-WT, DelEx5, C-terminus, or N-terminus. Data are presented as –fold change mean ± SD (n = 2–6)*.

* *Indicate P < 0.05*,

** *Indicate P < 0.01*,

*** *Indicate P < 0.001. n/a means data not available*.

N-terminus (0.2 μM) enhanced the secretion of RANTES 2.5, 4.5, and 3-fold at days 1, 7, and 14 respectively (*P* = 0.036, *P* = < 0.001, *P* = 0.003). The secretion of IP-10 was enhanced 5.5 and 2.7-fold at day 1 and 14, respectively (*P* = 0.003 and *P* = 0.037). N-terminus enhanced the secretion of MIP-1α 2.6-fold at day 14 (*P* = 0.004). The N-terminus also significantly enhanced secretion of IL-8 2.5-fold at day 3 (*P* = 0.038; Table 2).

The C-terminus and DelEx5 did not have any significant effects on the secretion of cytokines or chemokines, nor did they influence any differentiation markers tested.

### Differentiation of hMSC are stimulated by peptides derived by *exon 5* and *Q9NP70*

**Figure 3:**
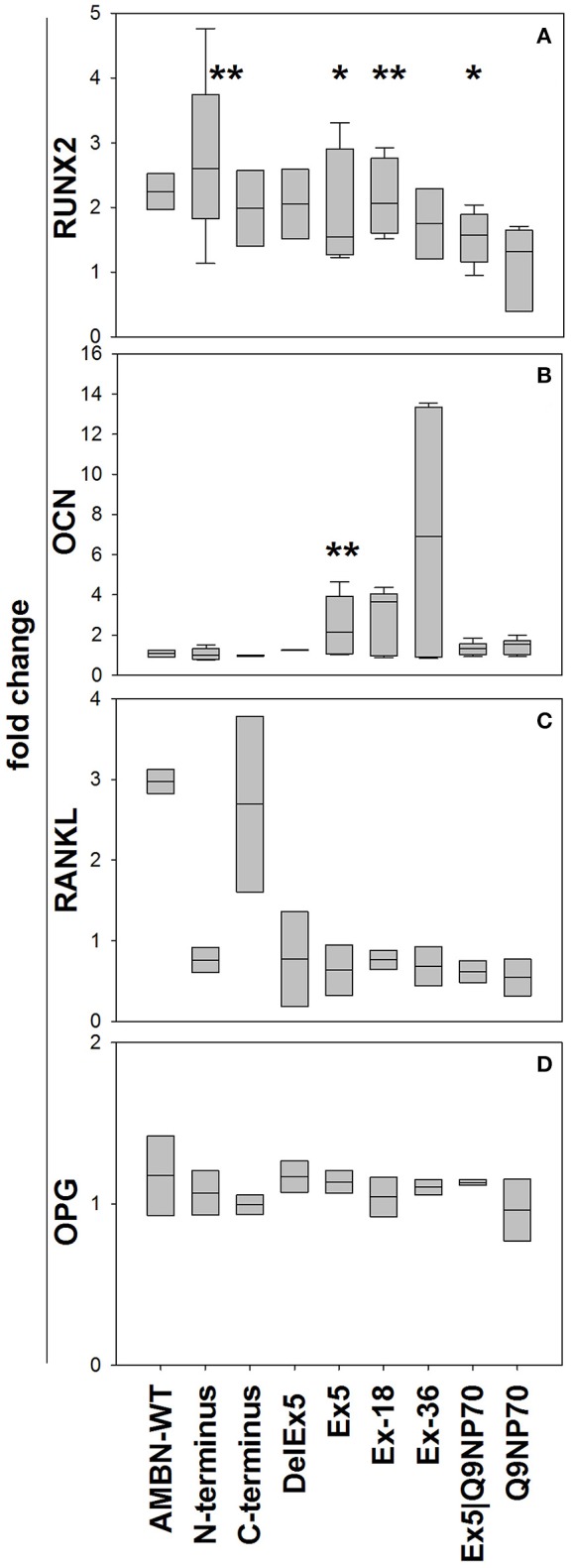
**Gene expression of RUNX2 (A)**, OCN **(B)**, RANKL **(C)**, and OPG **(D)** in human hMSCs at 72 h. Expression was normalized against the reference genes Glyceraldehyde-3-Phosphate Dehydrogenase (GADPH) and Beta Actin using the ΔΔCT method. Data are calculated relative to untreated control as fold-changes and presented as mean ± *SD* (*n* = 5) (%). ^*^Indicate *P* < 0.05, ^**^Indicate *P* < 0.01.

The N-terminus and the Ex5, Ex5-18, and Ex5|Q9NP70 peptides all stimulated the mRNA expression of RUNX2 (3-fold (*P* = 0.008), 1.9 -fold (*P* = 0.016), 2.1-fold (*P* = 0.004), and 1.5-fold (*P* = 0.019)), respectively (Figure 3A). Ex5 stimulated the mRNA expression of OCN 2.5-fold (*P* = 0.009; Figure 3B), however no significant effect was observed on the secretion of OCN to the cell culture medium (results not shown). AMBN-WT stimulated the mRNA expression of RANKL; however neither AMBN nor its fragments had any significant effect on mRNA expression of OPG at the time-point analyzed (Figures 3C,D, respectively).

### Mineralization of hMSC is mostly influenced by *exon 5* derived peptides

**Figure 4:**
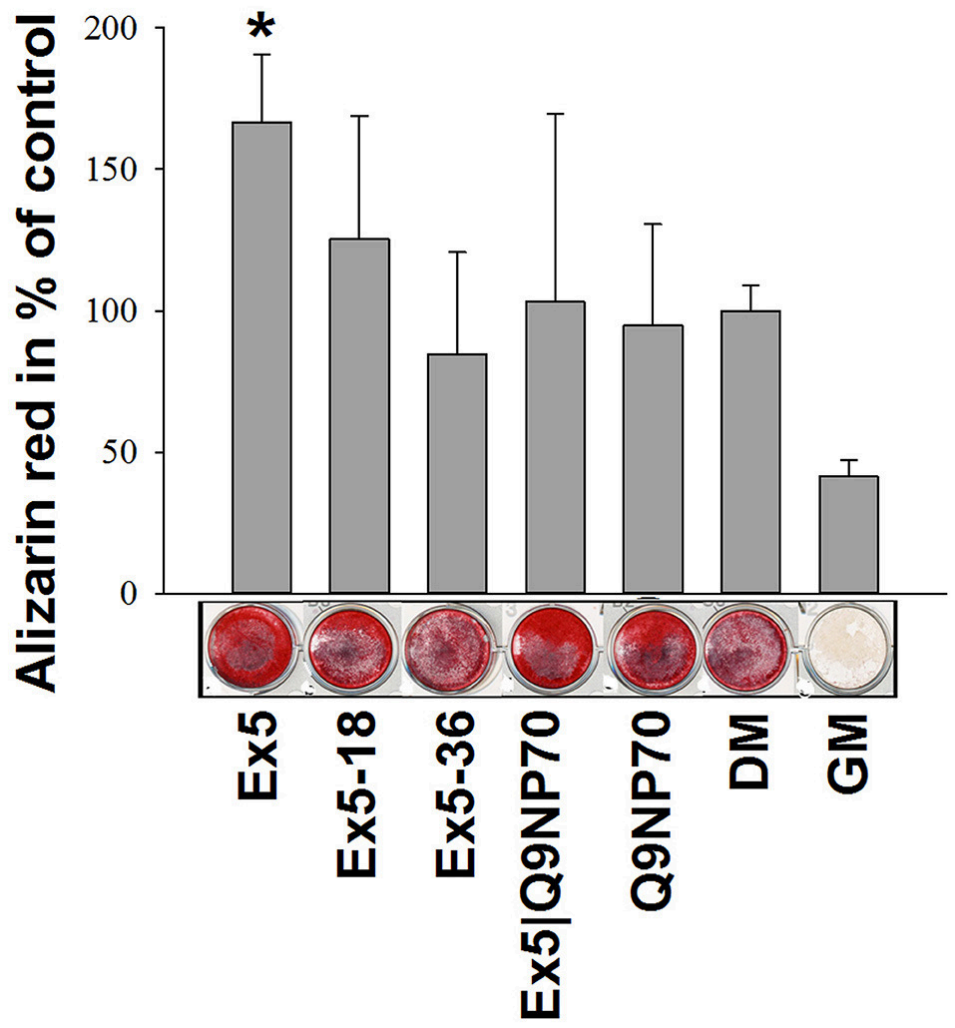
**Extracted alizarin red from mineralized surfaces of hMSC after 28 days incubation with Ex5, Ex5-18, Ex5-36, Ex5|Q9NP70, or Q9NP70 (0.2 μM)**. Data are presented as mean ± *SD* (*n* = 5) (%). ^*^Indicate *P* < 0.05.

In initial tests with hMSC growing in regular medium for 21 days, none of the larger fragments AMBN-WT, N-terminus, C-terminus, or DelEx5, promoted *in vitro* mineralization. However, *exon 5* and *Q9NP70* derived peptides had a visible but not statistically significant, effect on the formation of mineralized nodules in hMSC cell cultures (results not shown). Only when a combination of Ex5 peptide and osteogenic medium (DM) was used did the mineralization increase significantly to 1.7-fold over the DM-only control (*P* = 0.029; Figure 4).

## Discussion

AMBN is first and foremost an extra cellular matrix protein, and may as such constitute a slow-release depot for biological signals. AMBN has not been identified as an intact soluble protein *in vivo* (Brookes et al., 2001; Iwata et al., 2007), and no complete information is available on the processing of AMBN in tissues other than in teeth. In other tissues, fragments are probably released into solution from the self-assembled AMBN complex, but little is known about the nature and the function of these fragments. Here we have shown that some selected fragments have discrete and significant effects on cultured hMSC.

To be able to compare different peptides and fragments we performed the experiments with equimolar concentrations. The recombinant proteins and peptides were administered to hMSC in a dosage (0.2 μM) to reflect the levels previously shown to be secreted from cultured hMSC (Tamburstuen et al., 2011) and that has been shown to have effects in other experiments with hMSC (Tamburstuen et al., 2010). We also included a lower concentration of AMBN to test the responsiveness to concentration levels similar as found in mature osteoblasts (0.1 μM; Tamburstuen et al., 2011). Both concentrations induced effects on proliferation and secretion of chemokines, suggesting that the AMBN secreted from progenitor cells and osteoblasts (Tamburstuen et al., 2011) indeed can have an effect as a signaling molecule in mesenchymal tissues.

### Proliferation of hMSC were modulated by AMBN-WT and regions derived by *exon 5* and *Q9NP70*

Proliferation of stem cells is a key feature in healing and tissue homeostasis. Here we have shown that AMBN-WT and peptide Ex5 stimulate hMSC proliferation. In fact Ex5 alone is more efficient as a signal for proliferation for these cells than the whole WT molecule, most probably due to superior bioavailability and/or a more favorable structural conformation. Interestingly, when exon 5 was deleted out of the full length AMBN there was little effect on proliferation, suggesting that Ex5 is a key element for this process. This was further demonstrated by the fact that additional processing of Ex5 into smaller fragments minimizes the effect on proliferation. The other fragments tested here did not significantly alter proliferation of hMSC. This is also supported by other studies, where a 15 aa peptide derived by *exon 2* and *exon 3* was shown not to influence proliferation (Kitagawa et al., 2011, 2016). Moreover, overexpression of AMBN lacking *exon 5* and *exon 6* in mice resulted in reduced bone growth, femur length, and higher fracture rate (Lu et al., 2016a,b) indirectly suggesting the importance of *exon* 5 in cell proliferation.

The regions derived by *exon 5* and *exon 6* have been shown to be vital for proper development of enamel (Smith et al., 2009; Wazen et al., 2009). Interestingly, the upstream part of *exon* 6 encodes a peptide (Q9NP70) that was found to inhibit proliferation of hMSC. This inhibition is probably stronger than the positive signal from Ex5 since the combination of the two (Ex5|Q9NP70) has a net inhibitory effect. However, this may also be due to steric inhibition or interfering pathways. The *exon 6* derived Q9NP70 has been found *in vivo* during post-natal tooth development (Lee et al., 2003; Ravindranath et al., 2007) and thus may contribute by inhibiting cell proliferation at a stage where differentiation probably is more developmentally desired than proliferation.

The C-terminus fragment of AMBN showed no effect on hMSC proliferation. This fragment has however, been found to inhibit the proliferation of PDL and dental follicle cells (Zhang et al., 2011). This suggests that the effect of the various processed AMBN fragments might be tissue specific in real life situations.

### Cytokines/chemokines secretion enhanced by AMBN fragments

Several reports suggest that AMBN can play a role in regeneration of bone (Nakamura et al., 2006; Spahr et al., 2006; Tamburstuen et al., 2010; Lu et al., 2016a,b). Overexpression of AMBN has been found to increase osteoclastogenesis (Lu et al., 2013). Osteoclastogenesis and inflammation are both critical processes influencing the early stages in regeneration of bone (Mountziaris and Mikos, 2008). Here we have demonstrated that AMBN-WT significantly enhanced the secretion of MCP-1, IL-6, RANTES, MIP-1α, and IP-10. All these factors have been associated with osteoclastogenesis (Kotake et al., 1996; Watanabe et al., 2004; Kim et al., 2005, 2006) in addition to inflammatory processes (Schall et al., 1990; Dufour et al., 2002). Interestingly, again, this effect is only observed from peptides derived from the N-terminus part of the AMBN molecule. Moreover, when the N-terminus fragment was tested alone it also enhanced the secretion of IL-8.

None of the other fragments, DelEx5, C-terminus, Ex5, Ex5-18, Ex5-36, Ex5|Q9NP70, or Q9NP70, had any effect on the markers analyzed here. Relevant for bone formation is the fact that IL-8 so far is the only chemokine known to enhance secretion of MMPs (Li et al., 2003).

Both the N-terminus and AMBN-WT self-assemble into fibrils (Wald et al., 2013). It is a surprising but striking feature that only these fibril forming regions affected the secretion of cytokines / chemokines from hMSC. Receptor oligomerization (George et al., 2002) has been shown to enhance secretion of chemokines (Martinez-Martin et al., 2015). Fibril formation of AMBN may be a way to expose multiple signaling motifs in close proximity, which in turn could bind several motifs at once and thus initiating chemokine associated receptor oligomerization.

The AMBN-WT includes the C-terminus that has been suggested to provide cell anchoring (Fukumoto et al., 2004) through DGEA binding motifs (Cerný et al., 1996). AMBN has also been shown to bind integrin β1 (Iizuka et al., 2011; Lu et al., 2013), and Integrin β1 ligands have been shown to enhance secretion of RANTES (Peng et al., 2005). This may explain the observation that AMBN-WT is more potent in enhancing secretion of cytokines and chemokines than its processed products.

### Expressed markers for differentiation and mineralization

The expression of RUNX2, a transcriptional factor for extracellular matrix proteins like collagen and OCN (Ducy et al., 1997; Kern et al., 2001), has been found to be enhanced by AMBN and synthetic peptides derived from its 17 kDa fragment in PDL (Kitagawa et al., 2011, 2016). Enhanced hMSC expression of RUNX2 and OCN by the N-terminus, *exon 5*, and *Q9NP70* peptides presented here, support the idea that AMBN has effect on bone differentiation and mineralization.

This is further underlined by AMBN knockdown experiments that show reduction in alizarin red deposition during mineralized nodule formation (Iizuka et al., 2011). In the initial experiments on hMSC grown in GM we here found effects on mineralization only with the *exon 5* and the *Q9NP70* derivedpeptides. *Exon 5* and *exon 6* (including Q9NP70) have previously been shown to be important in enamel mineralization (Smith et al., 2009; Wazen et al., 2009). Accordingly, we confirmed that the Ex5 peptide stimulates calcium deposition under mineralizing conditions (DM) as visualized by alizarin staining.

## Conclusion

AMBN-WT enhances the proliferation of hMSCs and secretion of cytokines/chemokines. Moreover, the two main AMBN processing fragments and derived peptides have markedly diverse effects. The N-terminus portion seems to enhance secretion of cytokines/chemokines, whereas peptides derived by *exon 5* and *Q9NP70* modulate proliferation, enhance secretion of markers for hMSC differentiation and extracellular mineralization. In contrast the C-terminus fragment shows no discernible effect on hMSC differentiation or proliferation and is probably only involved in cell attachment.

## Author contributions

ØS contributed in experimental design, performed and analyzed experiments, drafted and wrote the manuscript, SL contributed in experimental design, drafting and finalizing the manuscript. JV provided essential materials and contributed in experimental design. JG contributed in experimental design and in drafting of the manuscript, JR contributed in experimental design, drafting and finalizing the manuscript.

### Conflict of interest statement

The authors declare that the research was conducted in the absence of any commercial or financial relationships that could be construed as a potential conflict of interest.
